# Go Slow, Mr. Secretary

**Published:** 1897-05

**Authors:** 


### EDITORIAL.

---

### GO SLOW, MR. SECRETARY.

SECRETARY WILSON, in listening to the spoilsmen of his party in their wail for public place, should occasionally step outside the atmosphere of Washington and learn the feelings of those who have welcomed the establishment of the merit-system in the Department of Agriculture as the wisest and most advanced movement ever inaugurated in that department of our government. He should know that the veterinary profession have spoken through their associations and organizations in behalf of the value of the merit-system of appointment. He only need ask of those who have entered this service by this method how much they appreciate its value and worth, and how many of them have only relinquished private practice to enter the Government service because of the feeling of security that the merit-system promised to those appointed under its rules. He only need seek for an expression of opinion from one and all of the veterinary colleges of North America, which have the true education of veterinarians at heart, to learn of the appreciation of the change inaugurated by Secretary Morton, and which, for the first time, gave impetus to a movement that found in the colleges an additional interest in governmental scientific work, and thus increased the special training required to more properly fit men for such work. He need only ask those whose positions will afford an unbiased expression of opinion as to the value of the work done to-day; the efficiency with which it is performed, and the character of the men in this branch of the public service as compared with the days of the "spoils system" in that department. Step into any former working centre of the department under the old system, and hear the stories of the good times when "party pull" was the entré and chain of security to place, and then compare it with one of these centres of work of to-day, where earnest, efficient attention to work and true scientific interest and zeal are displayed in the effort to discharge a sworn duty in the interest of the public service. You need not hide behind one requisition, Mr. Secretary, that brought you a youth for some service in which you preferred age and experience. Nor must this be a barrier for entrance to the public service, for the schools of to-day are equipping their scholars for these positions much better than the experience alone of the past twenty years has taught.

The service in this department was never better performed than during the past three years; the value of the work never so commensurate with the cost of the same. The extension of the work in every wise and profitable direction will find no opponents among those who have best studied its importance; but every step upon your part to return this department to the prey of the hungry horde of spoilsmen who follow in the wake of party success, and whose chief aim is to feed at the public crib, will bring only such results as will rob your administration of its high character, lower the standard of the work of your department, and lessen the interest of every true supporter of this work—collegiate, association, and individual. We sincerely trust that better judgment will prevail; and we repeat, "Go slow in this direction, Mr. Secretary."

